# An economic evaluation of community pharmacy–dispensed naloxone in Canada

**DOI:** 10.1177/17151635241228241

**Published:** 2024-02-13

**Authors:** Ashley Cid, Nikita Mahajan, William W.L. Wong, Michael Beazely, Kelly A. Grindrod

**Affiliations:** From the School of Pharmacy, University of Waterloo, Kitchener, Ontario

## Abstract

**Aims:**

To determine the cost-effectiveness of pharmacy-based intranasal (IN) and intramuscular (IM) naloxone distribution in Canada.

**Methods:**

We developed a state-transition model for pharmacy-based naloxone distribution, every 3 years, to illicit, prescription, opioid-agonist therapy and nonopioid use populations compared to no naloxone distribution. We used a monthly cycle length, lifetime horizon and a Canadian provincial Ministry of Health perspective. Transition probabilities, cost and utility data were retrieved from the literature. Costs (2020) and quality-adjusted life years (QALY) were discounted 1.5% annually. Microsimulation, 1-way and probabilistic sensitivity analyses were conducted.

**Results:**

Distribution of naloxone to all Canadians compared to no distribution prevented 151 additional overdose deaths per 10,000 persons, with an incremental cost-effectiveness ratio (ICER) of $50,984 per QALY for IM naloxone and an ICER of $126,060 per QALY for IN naloxone. Distribution of any naloxone to only illicit opioid users was the most cost-effective. One-way sensitivity analysis showed that survival rates for illicit opioid users were most influenced by the availability of either emergency medical services or naloxone.

**Conclusion:**

Distribution of IM and IN naloxone to all Canadians every 3 years is likely cost-effective at a willingness-to-pay threshold of $140,000 Canadian dollars/QALY (~3 × gross domestic product from the World Health Organization). Distribution to people who use illicit opioids was most cost-effective and prevented the most deaths. This is important, as more overdose deaths could be prevented through nationwide public funding of IN naloxone kits through pharmacies, since individuals report a preference for IN naloxone and these formulations are easier to use, save lives and are cost-effective. *Can Pharm J (Ott)* 2024;157:xx-xx.

## Introduction

The opioid crisis is one of the most serious recent public health crises in Canada, as over 40,000 apparent opioid toxicity deaths have occurred since January 2016.^1^ Canada’s opioid crisis is also a fentanyl crisis, which increases the risk of opioid-induced respiratory depression (OIRD).^2^ In 2022, 76% of opioid-related deaths involved fentanyl and 79% were from the unregulated supply.^1^ Naloxone, an antidote for OIRD, is available as a harm reduction tool in Canada in 2 dosage forms: an intramuscular (IM) injection ($30-$55 Canadian dollars [CAD]) and an intranasal (IN) spray ($150-$200 CAD).^3,4^ In Canada, health care is provincially managed, leading to disparities in naloxone access.^5^ Ontario, Quebec and the Northwest Territories offer publicly funded IN and IM naloxone kits, while all other provinces publicly fund only IM naloxone kits.^4,5^ Anecdotally, in provinces where only IM naloxone is publicly funded, informants acknowledge the difficulties in properly training and preparing the general public to prepare and administer IM naloxone, as it can be more stressful in an emergency situation in those who have less experience preparing and administrating injections.^4,5^ Across jurisdictions, naloxone eligibility includes current or past opioid use (prescription or unregulated), anyone at risk of OIRD, a contact of a person at risk or a person in a position to assist someone at risk.^3^ Naloxone programs, in general, are effective in preventing opioid-related fatalities.^6^ For example, a study in British Columbia estimated that the provincial naloxone program prevented 226 deaths between January and October 2016.^7^ An umbrella review also concluded that naloxone programs improve long-term knowledge about opioid overdoses, improve attitudes toward naloxone, provide sufficient training to effectively manage overdoses and effectively reduce opioid-related mortality.^8^

Community pharmacies are highly accessible primary health care sites located in urban, rural and remote areas of Canada, which increases access to publicly funded naloxone kits.^9^ The question of whether pharmacy-based naloxone kit distribution is cost-effective has not been studied in the Canadian context. A 2022 systematic review of economic evaluations involving community naloxone included 9 articles from the United States, which all concluded that community distribution of naloxone was cost-effective, with an incremental cost-utility ratio range of $58,738 to $111,000 US (2020) per quality-adjusted life year (QALY) gained.^10^ The review included only 1 study that examined pharmacy-based naloxone kit distribution.^11^ This study determined that one-time distribution of IN naloxone to high-risk prescription opioid patients prevented 14 additional overdose deaths per 100,000 persons, with an incremental cost-effectiveness ratio (ICER) of $56,699 US per QALY.^11^ There is a need to conduct a cost-effectiveness study of pharmacy-based naloxone in the Canadian context, as most studies have been conducted in the United States, which does not have a public health care funding model, and the expiry date of naloxone was recently extended to 3 years, which has not been included in other economic evaluations.^10-12^ The objective of this study was to determine the cost-effectiveness of pharmacy-based IM and IN naloxone distribution to consumers of prescription and illicit opioids, as well as opioid-agonist therapy (OAT) and nonopioid consumers (family or bystanders).

**Knowledge into Practice**

- Distribution of either intramuscular or intranasal naloxone to all Canadians every 3 years is cost-effective.
- More opioid-related deaths could be prevented through nationwide public funding of intranasal naloxone kits.
- Distribution of either format of naloxone to people who use illicit opioids was found to be the most cost-effective and prevented the most deaths.
- A universally accepted definition of “opioid overdose” is still needed. Future research should include identifying clear criteria for what can be considered a fatal or nonfatal opioid overdose event in community settings.
- Future research should also directly compare the efficacy between intramuscular and intranasal naloxone to allow for a direct comparison of cost-efficacy between the 2 formats instead of comparing to the status quo.

## Methods

### Overview and strategies

A state transition (Markov) model was used to perform a cost-utility analysis of both IM and IN naloxone distribution compared to the status quo, specifically:

1. (Status quo): IM or IN naloxone kits being distributed by any means other than a pharmacy
2. IM naloxone distributed by pharmacy together with any other means of distribution
3. IN naloxone distributed by pharmacy together with any other means of distribution

**Mise En Pratique Des Connaissances**

- La distribution de naloxone intramusculaire ou intranasale à tous les Canadiens tous les trois ans est rentable.
- Davantage de décès liés aux opioïdes pourraient être évités grâce au financement public national des trousses de naloxone intranasales.
- La distribution de l’un ou l’autre des formats de naloxone aux personnes qui consomment des opioïdes illicites s’est avérée la plus rentable et a permis de prévenir le plus grand nombre de décès.
- Une définition universellement acceptée de « surdose d’opioïdes » est encore nécessaire. Les recherches futures devraient comprendre l’identification de critères clairs pour ce qui peut être considéré comme un événement de surdose d’opioïdes mortel ou non mortel dans des environnements communautaires.
- Les recherches futures devraient également comparer directement l’efficacité entre la naloxone intramusculaire et intranasale afin de permettre une comparaison directe du rapport coût-efficacité entre les deux formats au lieu de la comparer au statu quo.

In the base-case analysis, we assess the distribution of IM or IN naloxone to all populations (illicit, prescription, OAT and nonopioid consumers) every 3 years vs status quo. Three scenario analyses were conducted to determine which population would benefit the most from naloxone distribution. The scenario analyses involved distributing IM or IN naloxone to only illicit, only OAT or only prescription opioid users compared to status quo. Although IM kits are less expensive, they require more training as well as a fee paid to the pharmacist for the training.^3^ IN naloxone kits require less training and are easier to use but are more expensive.^3^ The cost, markup, IM naloxone training fee and professional fees for both IM and IN naloxone were included in the analysis.^3,4^ A lifetime horizon was used, since the use of naloxone may be a recurring event.^11^ All health outcomes and costs (2020 CAD) were discounted at 1.5% annually, which is consistent with the 2017 Canadian guidelines for the Economic Evaluation of Health and Technologies.^13^ The analysis was carried out from a provincial Ministry of Health perspective, since naloxone is publicly funded.^4^ In the model, naloxone was distributed every 3 years based on its expiry date.^12^ The primary outcomes of this study were the total cost, total QALY and the ICER of both IN and IM naloxone dispensed through pharmacies. The secondary outcome was a prediction of the number of overdose deaths prevented because of IN and IM naloxone distribution.

### Cohort

The study cohort was derived from a population-based time-series study examining Ontario naloxone pharmacy program uptake that included OAT and prescription opioid recipients.^14^ Consistent with participants in the Ontario naloxone pharmacy program, we assumed an average age of 38 and equal distribution by sex.^14^ The proportion of illicit consumers in the Canadian population was not available in the literature; therefore, the proportion was estimated from a study of take-home naloxone dispensing to consumers of heroin in the United Kingdom.^15^ In the model, the remaining proportion of the population was assumed to have no/unknown opioid consumption, which may include past consumers of opioids or those who have never consumed any opioid.

### Decision model

**Figure 1:**
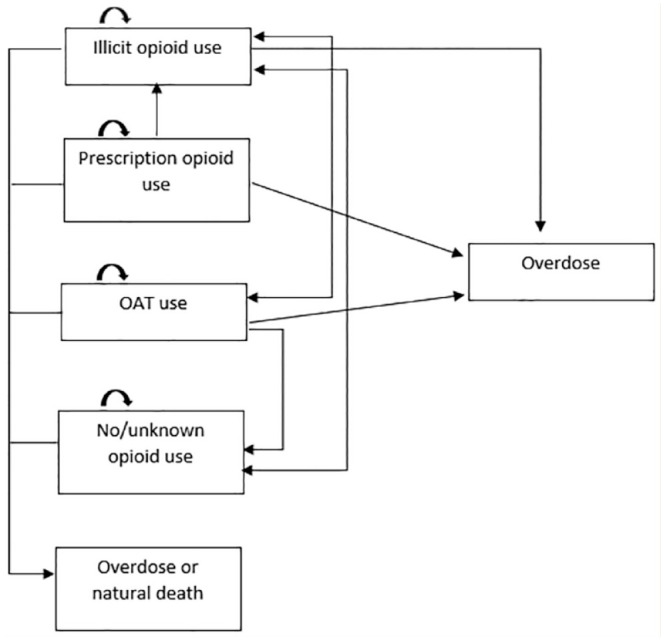
Markov model to demonstrate the movement of illicit, prescription, opioid-agonist therapy opioid consumers and nonopioid consumers between each other, with the probability for opioid overdose and/or natural death

**Figure 2:**
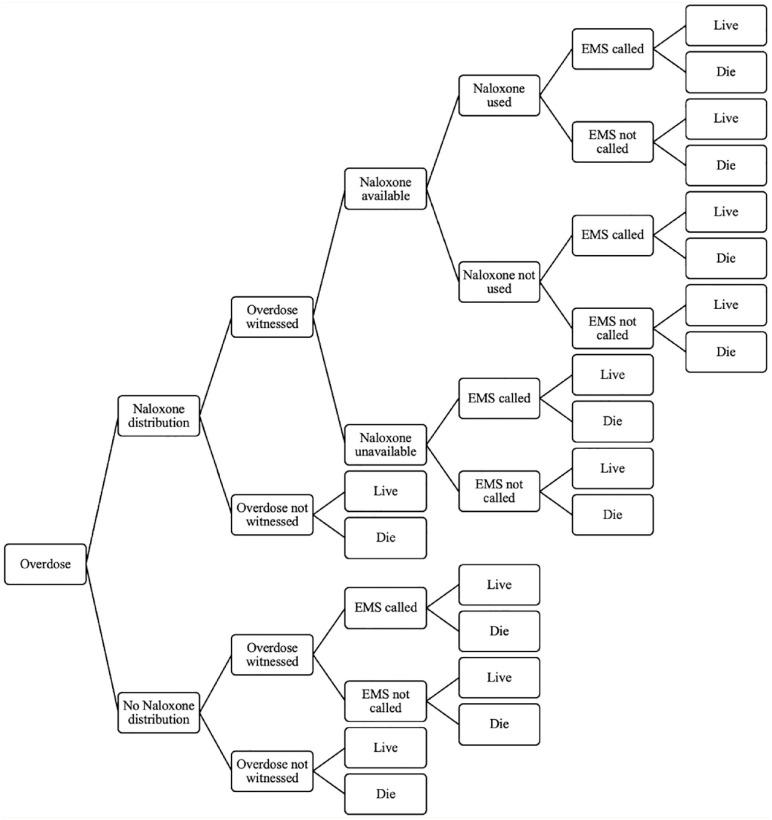
Decision tree to model the probability of surviving or dying from an opioid overdose once it occurs, which depends on naloxone distribution and availability, witnesses, emergency medical services being called and naloxone being used

TreeAge Pro 2021 decision analysis software was used to implement the state-transition (Markov) model (Figure 1) with the attached decision tree (Figure 2), to model the recursive course of an opioid overdose. The health states included in the model were consistent with the target populations described above. Since opioid consumption in any form can lead to overdose and/or death, the model allowed cohort members to move across the health state based on behaviour change or once an overdose occurred. The decision tree included the following events: naloxone distribution, the overdose being witnessed, naloxone being available, naloxone being used and emergency medical services (EMS) being called. Cohort members moved between predefined health states in monthly cycles until all members had died. The life table was used to model the probability of dying from natural causes for each of the arms.^16^

### Input data

Published literature was used to inform the transition probabilities and utilities data, including the range of values used in the model (Table 1). Canadian data were used where possible. The data for naloxone efficacy was gathered from the Ontario population-based study of naloxone program uptake.^14^ IN and IM naloxone were assumed to have the same efficacy, since data for their comparative efficacy do not exist. Cost data for naloxone, training and professional fees were taken from a study reporting the status of naloxone in Canada in 2020.^4^ Due to the lack of other Canadian cost data, all other cost data were assumed from the study conducted by Acharya et al.,^11^ as were the utilities for prescription opioid use, illicit opioid use and no illicit opioid use. Acharya et al.^11^ originally obtained the prescription opioid use utility from Hayes et al.,^17^ which studied health-related quality of life among chronic opioid users with chronic noncancer pain. The illicit opioid use utility and no illicit opioid utility were originally obtained from a study looking at joint health state utility estimators in substance use disorders, but Acharya et al.^11^ applied a 7% increase in utility for the no illicit opioid use state, following the approach taken by Coffin and Sullivan.^18,19^ The OAT opioid use utility was obtained from a study analyzing short-term health-related quality of life improvement during opioid agonist treatment.^20^ All cost data were converted to 2020 CAD using Purchasing Power Parities and the Statistics Canada Consumer Price Index.^21,22^ The proportion of individuals who survived a witnessed overdose in the illicit and OAT arms was estimated from the expert opinion of MB due to the lack of available data. Other specific assumptions included that every person in the model received a naloxone kit and due to the lack of data, illicit users could not transition back to prescription use and prescription opioid users would not stop opioid use.

**Table 1:** Model input data

Input parameters	Base-case mean (range)	Source
Baseline proportion illicit opioid users	0.30 (0.05-0.6)	Langham et al.^15^
Baseline proportion prescription users	0.192 (0.144-0.24)	Antoniou et al.^14^
Baseline proportion OAT users	0.18 (0.135-0.225)	Antoniou et al.^14^
Baseline proportion of nonopioid users	0.328	Assumption
**Markov transition probabilities (monthly)**		
Illicit opioid use to no opioid use	0.0073 (0.00549-0.0091)	Luchenski et al.^23^
Illicit opioid use to overdose	0.008 (0.006-0.01)	Acharya et al.^11^
Prescription opioid use to illicit opioid use	0.0062 (0.0047-0.0078)	Cicero et al.^24^
Prescription opioid use to overdose	1.79E-6 (1.34E-6 to 2.23E-6)	Cho et al.^25^
OAT use to overdose	0.00258 (0.001935-00322)	Socías et al.^26^
OAT to illicit opioid use	0.135 (0.099-0.169)	Dong et al.^27^
Illicit opioid use to OAT use	0.706 (0.53-0.88)	Socías et al.^28^
OAT use to no opioid use	0.138 (0.1-0.17)	Socías et al.^28^
**Illicit opioid overdose decision tree**		
Proportion of overdose witnessed	0.95 (0.71-1.19)	Somerville et al.^29^
Proportion of overdose not witnessed and survive	0.6 (0.45-0.75)	Assumption
Probability of naloxone being available	0.852 (0.639-1.065)	Moustaqim-Barrette et al.^30^
Probability of naloxone being used	0.75 (0.56-0.94)	Somerville et al.^29^
Probability of EMS being called when naloxone is used	0.66 (0.495-0.825)	Buresh et al.^31^
Probability of EMS being called when naloxone is not used or available	0.33 (0.29-0.50)	Uyei et al.^32^
Proportion who survive overdose (without naloxone and EMS)	0.76 (0.68-0.84)	Acharya et al.^11^
Relative increase in survival with the use of naloxone or EMS	0.96 (95.5-97.1)	Antoniou et al.^14^
**Prescription opioid overdose decision tree**		
Proportion of overdose witnessed	0.72 (0.32-0.94)	Acharya et al.^11^
Proportion of overdose not witnessed and survive	0.9 (0.80-0.94)	Langham et al.^15^
Probability of naloxone being available	0.0029 (0.0022-0.0031)	Antoniou et al.^14^
Probability of naloxone being used	0.556 (0.417-0.695)	Antoniou et al.^14^
Probability of EMS being called when naloxone is used or available	0.74 (0.55-0.95)	Acharya et al.^11^
Probability of EMS being called when naloxone is not used	0.38 (0.30-0.80)	Acharya et al.^11^
Proportion who survive overdose (without naloxone and EMS)	0.88 (0.80-0.97)	Acharya et al.^11^
Relative increase in survival with the use of naloxone or EMS	0.96 (95.5-97.1)	Antoniou et al.^14^
**OAT overdose decision tree**		
Proportion of overdose witnessed	0.773 (0.580-0.966)	Siegler et al.^33^
Proportion of overdose not witnessed and survive	0.8 (0.6-1.0)	Assumption
Probability of naloxone being available	0.053 (0.040-0.066)	Antoniou et al.^14^
Probability of naloxone being used	0.744 (0.558-0.93)	Siegler et al.^33^
Probability of EMS being called when naloxone is used or available	0.563 (0.422-0.703)	Jakubowski et al.^34^
Probability of EMS being called when naloxone is not used	0.38 (0.30-0.80)	Acharya et al.^11^
Proportion who survive overdose (without naloxone and EMS)	0.88 (0.80-0.97)	Acharya et al.^11^
Relative increase in survival with the use of naloxone or EMS	0.96 (95.5-97.1)	Antoniou et al.^14^
**Cost (2019 Canadian dollars)**		
Intramuscular naloxone	47.50 (35-59)	So et al.^4^
Intranasal naloxone	183 (137-228)	So et al.^4^
Pharmacist training fee	8.89 (6-11)	So et al.^4^
EMS visit	2632 (1974-3290)	Acharya et al.^11^
EMS transport to hospital	443 (332-553)	Acharya et al.^11^
Emergency department care	1301 (975-1626)	Acharya et al.^11^
Prescription opioid use	1292 (969-1615)	Acharya et al.^11^
OAT opioid use	1292 (969-1615)	Acharya et al.^11^
Illicit opioid use	1901 (1425-2376)	Acharya et al.^11^
No illicit opioid use	794 (595-992)	Acharya et al.^11^
**Utilities (0 = death; 1 = perfect health)**		
Prescription opioid use	0.68 (0.48-0.88)	Acharya et al.^11^
Illicit opioid use	0.56 (0.39-0.73)	Acharya et al.^11^
No illicit opioid use	0.60 (0.42-0.78)	Acharya et al.^11^
OAT opioid use	0.59 (0.44-0.74)	Nosyk et al.^20^

EMS, emergency medical services; OAT, opioid-agonist therapy.

### Analytical plan

Measurable outcomes included the total cost of each strategy, total QALYs and the ICERs. The willingness-to-pay (WTP) threshold is referenced from the World Health Organization, which is defined as 3 times the gross domestic product (GDP) per capita, or $140,000 (based on the 2019 Canadian GDP, available at the time of model development).^35,36^ A life year (LY) analysis was also conducted due to recent criticism of using utility assumptions when calculating quality-adjusted life years (Appendix 1, available in Supplementary Material section online).^37^ Microsimulation was used to determine the number of overdose deaths prevented. We performed a probabilistic sensitivity analysis with 5000 samples of 100,000 individual trials to evaluate the sensitivity of outcomes to uncertainty in input parameters. If a range of values was not available, a 25% change from the base-case value was used. All probabilistic parameters and utilities used in the model were represented by beta distributions, and all cost parameters were represented by gamma distributions formed by the corresponding ranges. One-way sensitivity analysis to assess the robustness of the model was completed, with the results reported as a tornado diagram (Appendices 2 and 3, available in Supplementary Material section online), using the top 10 most influential parameters. The model was validated by an expert in the harm reduction field.

## Results

### Base-case and scenario analyses

In the model’s base-case scenario, distribution of either IM or IN naloxone kits every 3 years to all populations prevented 151 additional overdose deaths per 10,000 individuals (Table 2). If naloxone was distributed to only illicit, prescription or OAT populations, it prevented 287,247 and 141 additional overdose deaths per 10,000 individuals, respectively. The total costs for IM naloxone were slightly lower than IN naloxone, $328,974 vs $330,619 CAD. Both IM and IN naloxone were cost-effective at a WTP threshold of $140,000 per QALY, as the ICER for IM naloxone was $50,984 per QALY ($30, 464 per LY) and $126,060 per QALY ($75,323 per LY) for IN naloxone, compared to the status quo. When distributing to illicit consumers, the ICER was $35,717 per QALY ($21,346 per LY) for IM naloxone and $74,447 per QALY ($44,492 per LY) for IN naloxone when comparing both to the status quo. When distributing to prescription consumers, the ICER was $44,944 per QALY ($26,839 per LY) for IM naloxone and $104,051 per QALY ($62,135 per LY) for IN naloxone compared to the status quo. When distributing to OAT consumers, the ICER was $50,398 ($30,120 per LY) per QALY for IM naloxone and $124,505 per QALY ($74,408 per LY) for IN naloxone, compared to the status quo. Overall, the 2 most cost-effective groups of consumers to distribute either IM or IN naloxone to are illicit and prescription opioid users. The highest QALY occurred with prescription users at 19.76, while the QALY for illicit users was 18.79 and 19.06 for OAT users.

**Table 2:** Base-case and scenario analyses results to demonstrate total costs, total QALYs, ICERs and additional overdose deaths prevented when IM or IN naloxone is distributed to everyone and only to illicit, prescription and OAT users

Scenarios	Total cost ($)	Total QALYs	Incremental cost (CAD)	Incremental QALY	ICER (CAD/QALY)*	Additional overdose deaths prevented (per 10,000)
Base-case scenario						
No pharmacy naloxone distribution	327,857	19.20	—	—	—	—
IM naloxone distribution	328,974	19.23	1117	0.0219	50,984	151
IN naloxone distribution	330,619	19.23	2762	0.0219	126,060	151
Naloxone distribution to only illicit opioid users						
No pharmacy naloxone distribution	320,515	18.75	—	—	—	—
IM naloxone distribution	322,014	18.79	1499	0.042	35,717	287
IN naloxone distribution	323,640	18.79	3125	0.042	74,447	287
Naloxone distribution to only prescription opioid users						
No pharmacy naloxone distribution	383,673	19.73	—	—	—	—
IM naloxone distribution	384,919	19.76	1246	0.0277	44,944	247
IN naloxone distribution	386,558	19.76	2885	0.0277	104,051	247
Naloxone distribution to only OAT users						
No pharmacy naloxone distribution	316,400	19.04	—	—	—	—
IM naloxone distribution	317,518	19.06	1118	0.0222	50,398	141
IN naloxone distribution	319,161	19.06	2761	0.0222	124,505	141

CAD, Canadian dollars; ICER, incremental cost-effectiveness ratio; IM, intramuscular; IN, intranasal; OAT, opioid-agonist therapy; QALY, quality-adjusted life year.

* Compared to status quo.

### One-way sensitivity analysis

The most influential parameters for the base-case analysis involving only IN naloxone distribution (Appendix 2, available in Supplementary Material section online) or only IM naloxone (Appendix 3, available in Supplementary Material section online) were 1) the survival rate of illicit opioid consumers with EMS at the scene (illicit_EMS_live), 2) illicit opioid consumers having naloxone administered and having EMS at the scene (illicit_N_EMS_live) and 3) the survival rate of illicit opioid consumers without naloxone or EMS at the scene (illicit_noEMS_live). Other highly influential parameters included the total proportion of illicit opioid consumers, the health utility of no illicit opioid consumption and the relative increase in survival with the use of naloxone (naloxone efficacy).

### Probabilistic sensitivity analysis

See Appendices 4 and 5, available in Supplementary Material section online, for the cost-effectiveness scatterplot highlighting IN naloxone and IM naloxone compared to no pharmacy naloxone distribution, respectively. With a WTP at $140,000 per QALY, IN naloxone is 50% cost-effective and IM naloxone is 58% cost-effective. As WTP increases, the cost efficacy also gradually increases. When the WTP is $50,000 per QALY, IN naloxone is 25% cost-effective and IM naloxone is 49% cost-effective. At a WTP of $100,000 per QALY, IN naloxone is 44% cost-effective and IM naloxone is 56% cost-effective.

## Discussion

The analysis showed that the distribution of IM and IN naloxone through pharmacies led to modest reductions in opioid overdose deaths. Both IM and IN naloxone would be cost-effective at a WTP threshold of $140,000 CAD per QALY, when distributed every 3 years. However, in Canada, there is another conventional WTP threshold of $50,000 from the Canadian Agency for Drugs and Technologies in Health (CADTH).^38^ If this WTP threshold were to be used in this analysis, then only IM naloxone would be considered cost-effective if distributed every 3 years. However, since there is no absolute ruling on which WTP threshold should be used in Canada, using either WTP may be considered acceptable.^39^ The probabilistic sensitivity analysis indicated that both IM and IN naloxone would be cost-effective at $140,000 per QALY 58% and 50% of the time, respectively. In our model, limiting distribution to illicit opioid users was the most cost-effective scenario. When compared to the study by Acharya et al.,^11^ they showed that IN naloxone distribution to both illicit opioid users and prescription opioid users was cost-effective. For example, we determined that one-time distribution of IN naloxone had an ICER of $56,699 US per QALY, but Acharya et al.’s^11^ ICER was representative of distribution to both prescription and illicit opioid users, while we also included distribution to OAT users and bystanders. The model by Acharya et al.^11^ also reported a similar level of uncertainty for IN naloxone, as they found that a one-time and biannual IN naloxone distribution in the United States would be cost-effective 29% and 50% of the time, respectively, with a WTP of $100,000 USD. While our model assumed that illicit opioid users only consumed heroin (due to the challenge in assessing the cost-effectiveness of naloxone in the context of fentanyl), Acharya et al.^11,40^ included some fentanyl proportion data based on a single mixed-methods study conducted in Rhode Island. This study found that 50% of heroin users had an apparent overdose with fentanyl.^40^ Canada is missing data for the total proportion of the population that consumes heroin and the total proportion of the population that consumes fentanyl. The addition of illicit fentanyl consumption data to the model would likely and possibly significantly further improve the cost-effectiveness of naloxone.

A systematic review also identified that most naloxone economic evaluations to date have focused on a single population, whereas most pharmacy naloxone programs include multiple target populations.^10^ For example, Townsend et al.^41^ completed an economic analysis of naloxone distribution to first responders and laypersons. They determined that distribution would prevent 21% of overdose deaths and reported an incremental cost-utility ratio (ICUR) of $13,568 to $16,907 US per QALY.^41^ Another study reported that naloxone distribution to heroin consumers in the United Kingdom would reduce overdose deaths by 6.6% and had an ICUR of £1312 per QALY.^15^ Similarly, an economic evaluation of naloxone distribution in Scotland to individuals at risk of opioid overdose following prison release found a 3.5% decrease in deaths with an ICUR of £23,209 per QALY.^42^ All ICURs are lower than what was reported in our model, but our study considered the distribution of both IM and IN naloxone, to a broad population, with recurrent distribution every 3 years.

Of note, the total QALYs were higher every time naloxone was distributed in our model. This aligns with the concept that naloxone is effective at preventing opioid overdose deaths and would improve the quality of life for someone who receives it when experiencing an opioid overdose.^6^ In the 1-way scenario analysis, for both IM and IN naloxone, the model was most sensitive to the survival rate (i.e., if more people survive with naloxone, it is more cost-effective). The proportion of illicit opioid consumers was also influential, as they have higher rates of overdose and lower rates of survival and are more likely to benefit from naloxone.^2^ If the proportion of illicit users were higher, or if fentanyl use were considered along with heroin use, it is likely the model would be even more cost-effective. Another influential parameter found was the efficacy of naloxone. The more efficacious naloxone is, the more cost-effective it becomes, which is a factor to consider if the effectiveness of naloxone decreases (i.e., if more potent opioids/fentanyl analogues become more prevalent).

### Limitations

This research has several limitations. First, if Canadian-specific parameters could not be found, data were gathered from the international literature or by relying on experts in the field. For example, the proportion of overdoses not witnessed where the individual survives, for both illicit and OAT users, is not available in the literature. There is currently no agreed-upon definition for a nonfatal opioid overdose, whether measured by self-report, clinical data or administrative data.^43^ Future research should identify criteria used as a marker of both fatal and nonfatal opioid overdose events to establish a universally accepted definition. Contrary to standard best practice in health economics, this model was not able to directly compare the cost-efficacy between IM and IN naloxone, as there are no comparative efficacy data. Therefore, instead of comparing both formats to each other, they were compared to the status quo. Future research should investigate the direct efficacy of IM and IN naloxone, so that future studies can directly compare the 2 formats. Our model assumes a distribution of every 3 years based on the expiry date of naloxone provided by the manufacturer, but naloxone has been proven to work well beyond its expiration date and therefore may not always be accessed every 3 years.^12,44^ Our model also does not account for patients who have used naloxone and return earlier than 3 years to receive a replacement kit. Likewise, other individuals, particularly prescription opioid consumers who are offered naloxone kits, may not accept a kit if offered one due to the stigma surrounding its use, which has been cited as a barrier to wider uptake.^45^

This study aligns well with the recent Canadian national consensus guidelines on naloxone prescribing for health care providers, which states that to optimize naloxone distribution and curb the opioid crisis, naloxone should be offered to all patients with an opioid prescription at the pharmacy.^46^ In addition, the population that consumes illicit opioids would also benefit from optimal distribution of naloxone. Based on this study, providing naloxone to prescription and illicit opioid consumers in either format would be cost-effective. Of note, dispensing naloxone is not the only solution to curb the opioid crisis; a recent population-based study mentioned that the naloxone program alone is insufficient to curb opioid overdose deaths, but a combination of multiple harm reduction methods is needed.^47^

## Conclusion

The findings from this study demonstrate that community pharmacy distribution of publicly funded IM and IN naloxone in Canada is a worthwhile investment that can and should be implemented across Canada, particularly to the population of Canadians who consume illicit opioids. The results of the study may be used to support policymaking about naloxone programs, including whether to expand the distribution of publicly funded IN naloxone across Canada. Since IN naloxone is anecdotally the preferred method for naloxone administration by the general public, publicly funded IN naloxone across Canada could increase uptake of naloxone in these populations and thereby optimize naloxone distribution. Future research is needed to identify the proportion of the population and the rate of overdose incidence and death from new and emerging opioids such as fentanyl and carfentanil, as well as in cases when benzodiazepines are consumed with opioids. In addition, future efforts should be made to educate pharmacy staff and patients about naloxone-related stigma and how to overcome it using effective and open communication as well as education. ■

## Supplemental Material

sj-docx-1-cph-10.1177_17151635241228241 – Supplemental material for An economic evaluation of community pharmacy–dispensed naloxone in Canada Supplemental material, sj-docx-1-cph-10.1177_17151635241228241 for An economic evaluation of community pharmacy–dispensed naloxone in Canada by Ashley Cid, Nikita Mahajan, William W.L. Wong, Michael Beazely and Kelly A. Grindrod in Canadian Pharmacists Journal / Revue des Pharmaciens du Canada
