# Supplementary material for: An economic evaluation of community pharmacy–dispensed naloxone in Canada
Source: Can Pharm J (Ott). 2024 Feb 13;157(2):84–94. doi: 10.1177/17151635241228241 (PMC10924576; doi:10.1177/17151635241228241)

**APPENDIX**

**Appendix 1. Base case and scenario analyses results to demonstrate total costs, total LYs, ICERs, and additional overdose deaths prevented when IM or IN naloxone is distributed to everyone, and only to illicit, prescription and OAT users.**

| **Scenarios** | **Total Cost ($)** | **Total LYs** | **Incremental Cost (CAD)** | **Incremental LY** | **ICER (CAD/LY)**  *****Compared to status quo | **Additional overdose deaths prevented (per 10,000)** |
| --- | --- | --- | --- | --- | --- | --- |
| *Base case scenario* | | | | | | |
| No Pharmacy Naloxone Distribution | 327 857 | 31.83 | - | - | - | - |
| IM Naloxone Distribution | 328 974 | 31.86 | 1117 | 0.0367 | 30 464 | 151 |
| IN Naloxone Distribution | 330 619 | 31.86 | 2762 | 0.0367 | 75 323 | 151 |
| *Naloxone distribution to only illicit opioid users* | | | | | | |
| No Pharmacy Naloxone Distribution | 320 515 | 31.36 | - | - | - | - |
| IM Naloxone Distribution | 322 014 | 31.43 | 1499 | 0.070 | 21 346 | 287 |
| IN Naloxone Distribution | 323 640 | 31.43 | 3125 | 0.070 | 44 492 | 287 |
| *Naloxone distribution to only prescription opioid users* | | | | | | |
| No Pharmacy Naloxone Distribution | 383 673 | 31.70 | - | - | - | - |
| IM Naloxone Distribution | 384 919 | 31.74 | 1246 | 0.0464 | 26 839 | 247 |
| IN Naloxone Distribution | 386 558 | 31.74 | 2885 | 0.0464 | 62 135 | 247 |
| *Naloxone distribution to only OAT users* | | | | | | |
| No Pharmacy Naloxone Distribution | 316 400 | 31.80 | - | - | - | - |
| IM Naloxone Distribution | 317 518 | 31.83 | 1118 | 0.0371 | 30 120 | 141 |
| IN Naloxone Distribution | 319 161 | 31.83 | 2761 | 0.0371 | 74 408 | 141 |

**Appendix 2. Tornado diagram for top ten influential parameters – IN naloxone distribution only**


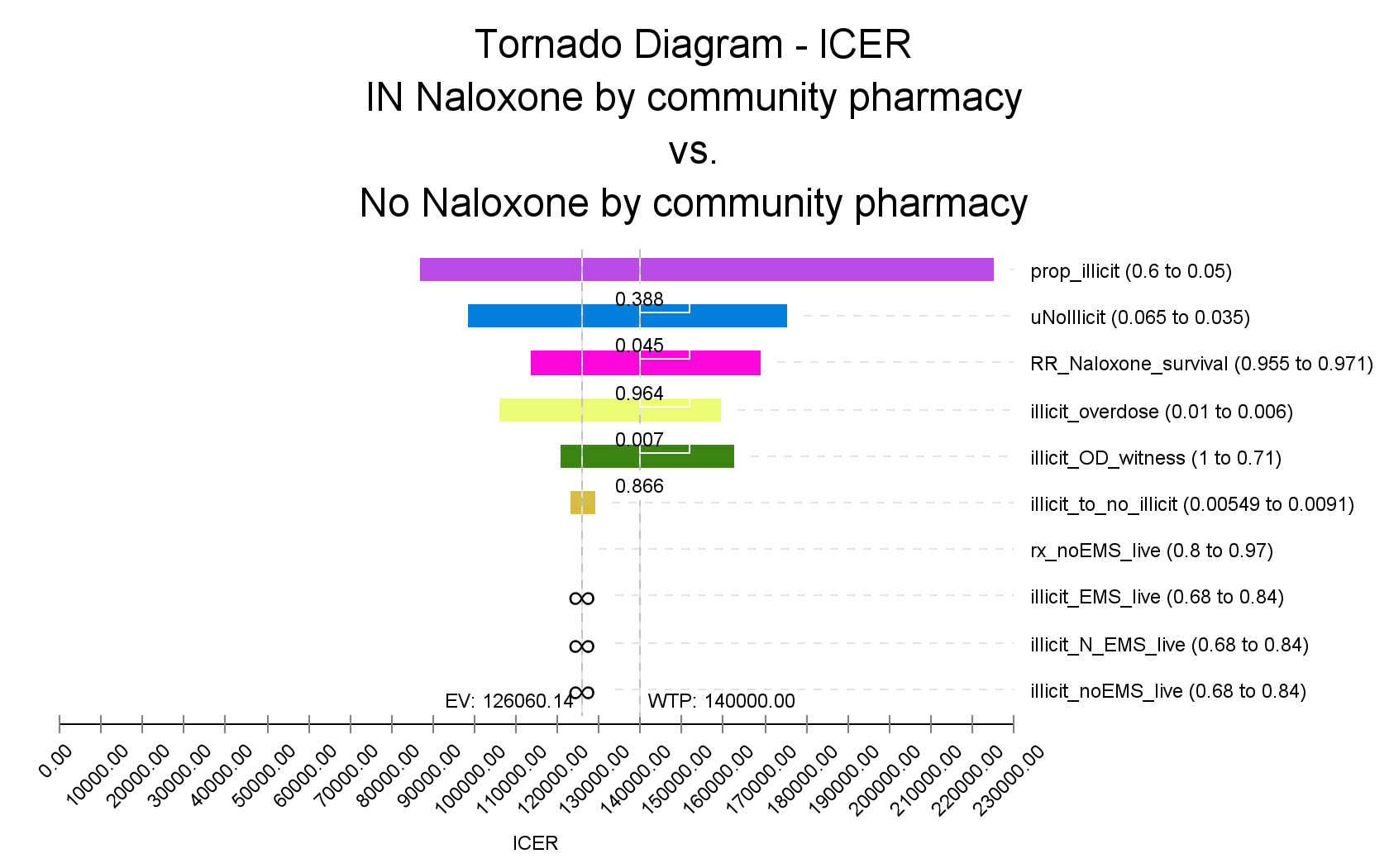


OD= Overdose, N= Naloxone, EMS= Emergency Medical Services, ICER= Incremental Cost Effectiveness Ratio, EV= Expected Value, RR= Relative risk, rx = prescription users.

**Appendix 3. Tornado diagram for top ten influential parameters – IM naloxone distribution only**


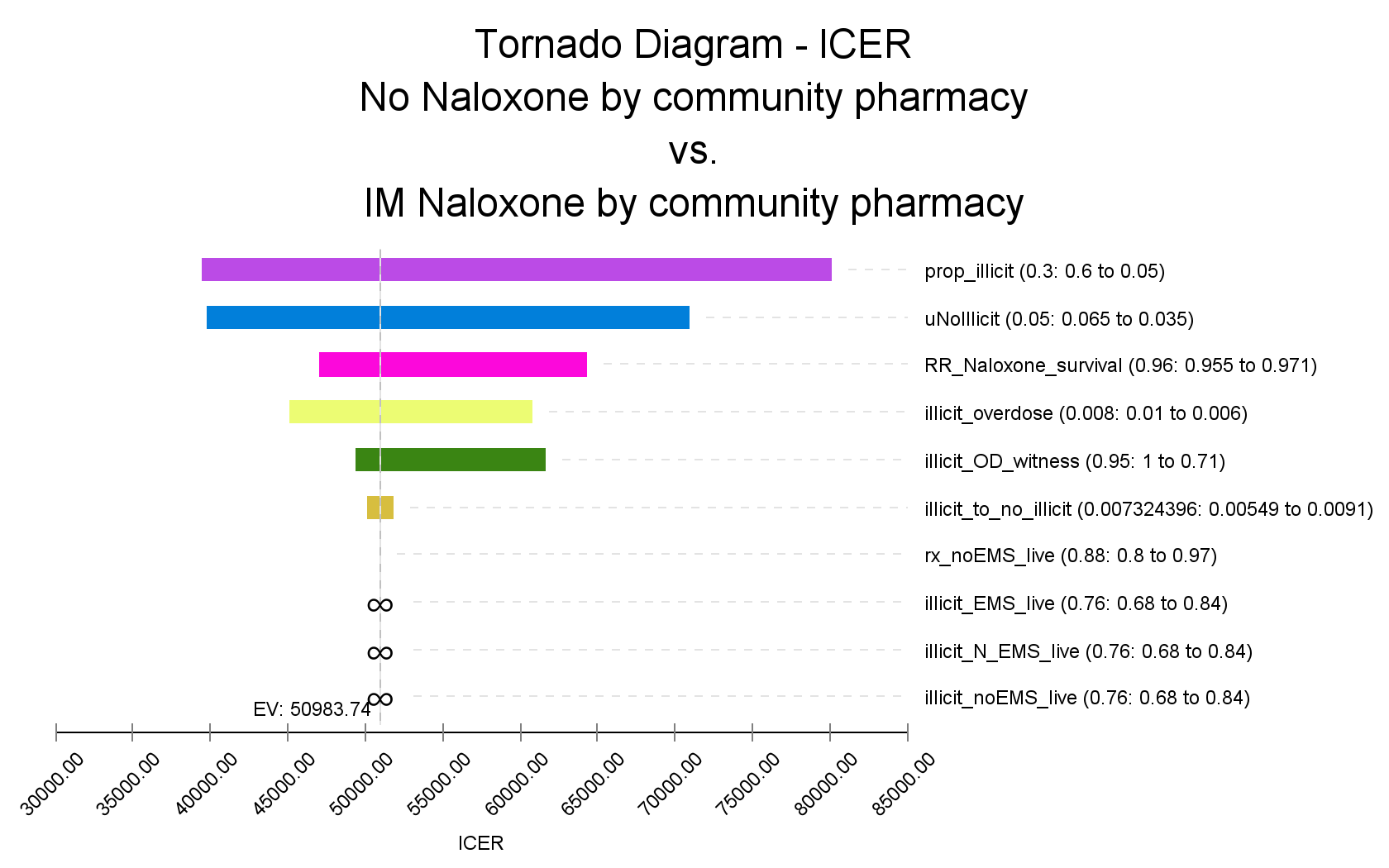


OD= Overdose, N= Naloxone, EMS= Emergency Medical Services, ICER= Incremental Cost Effectiveness Ratio, EV= Expected Value, RR= Relative risk, rx = prescription users.

**Appendix 4. Cost-Effectiveness Scatterplot – IN naloxone**


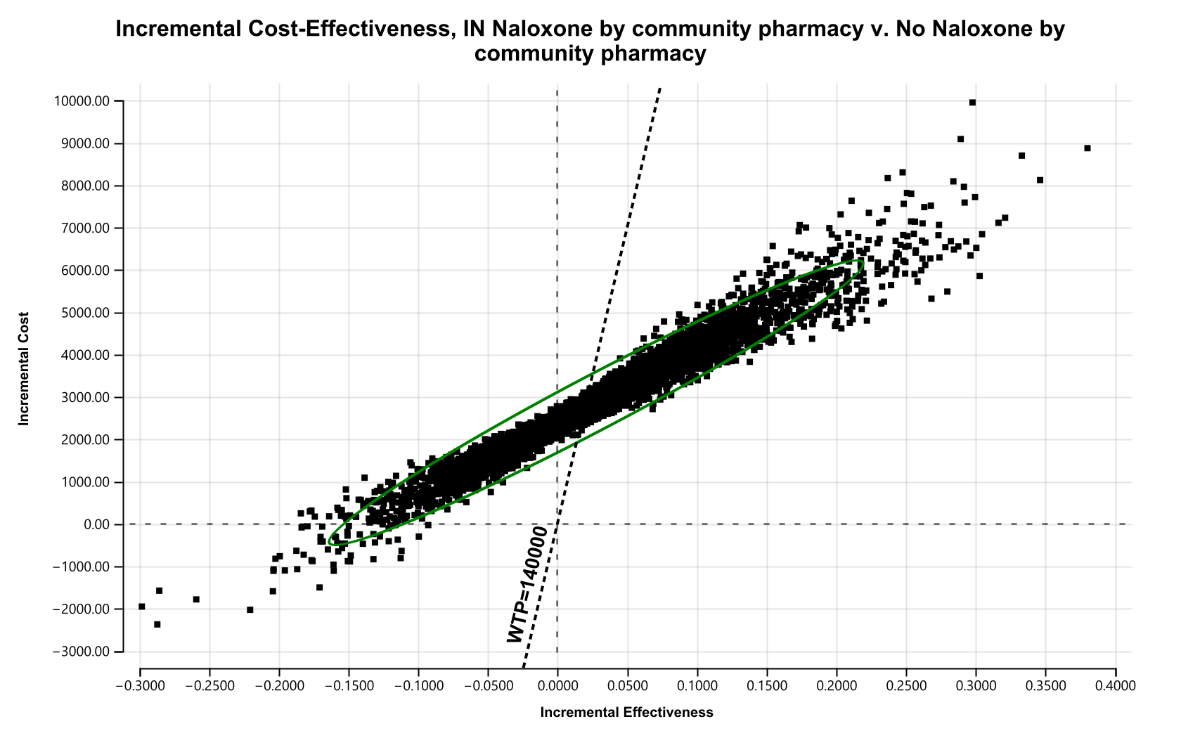


**Appendix 5. Cost-Effectiveness Scatterplot – IM naloxone**


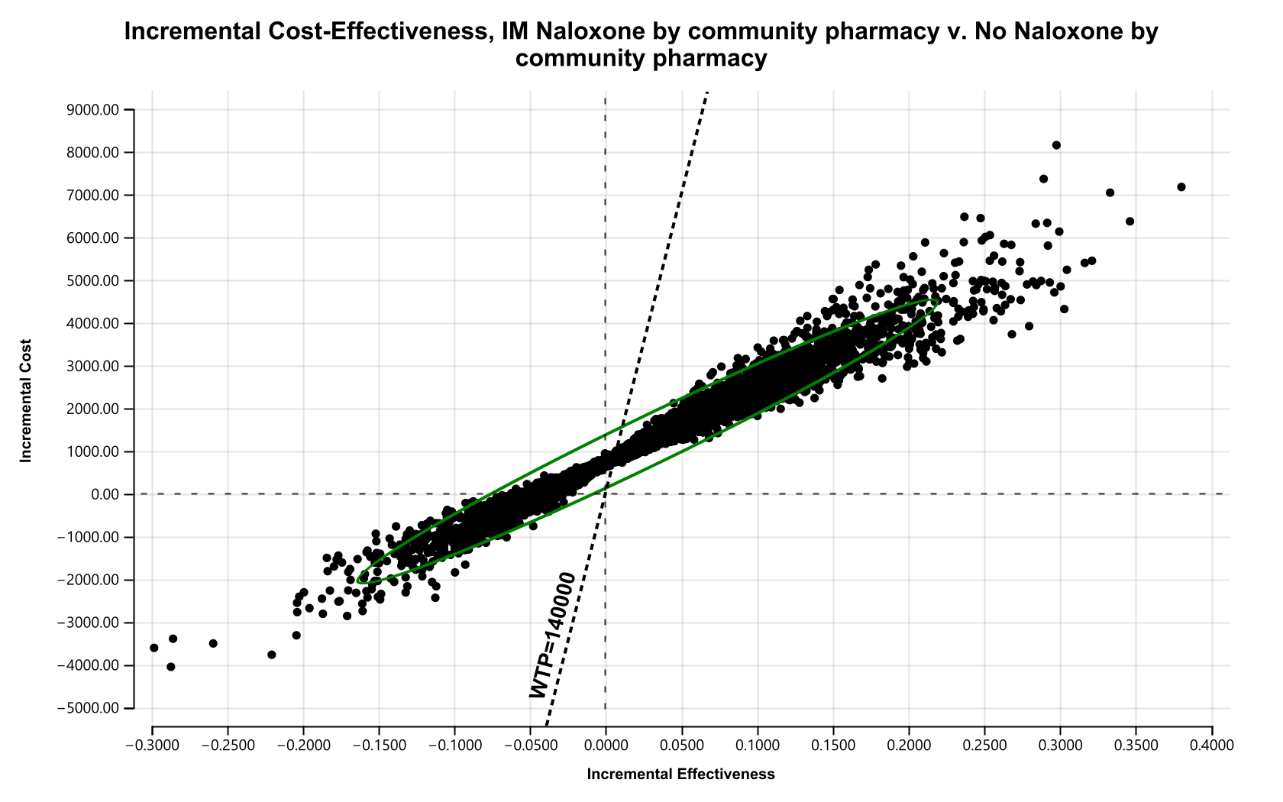

Supplement: sj-docx-1-cph-10.1177_17151635241228241 – Supplemental material for An economic evaluation of community pharmacy–dispensed naloxone in Canada [file sj-docx-1-cph-10.1177_17151635241228241.docx]
